# Identification of Pharmacophoric Fragments of DYRK1A Inhibitors Using Machine Learning Classification Models

**DOI:** 10.3390/molecules27061753

**Published:** 2022-03-08

**Authors:** Mengzhou Bi, Zhen Guan, Tengjiao Fan, Na Zhang, Jianhua Wang, Guohui Sun, Lijiao Zhao, Rugang Zhong

**Affiliations:** 1 Key Laboratory of Environmental and Viral Oncology, College of Life Science and Chemistry, Faculty of Environment and Life, Beijing University of Technology, Beijing 100124, China; bimengzhou@emails.bjut.edu.cn (M.B.); fannie818@126.com (T.F.); sunguohui@bjut.edu.cn (G.S.); zhaolijiao@bjut.edu.cn (L.Z.); lifesci@bjut.edu.cn (R.Z.); 2 Beijing Municipal Key Laboratory of Child Development and Nutriomics, Translational Medicine Laboratory, Capital Institute of Pediatrics, Beijing 100020, China; mengai518@126.com; 3 Department of Medical Technology, Beijing Pharmaceutical University of Staff and Workers, Beijing 100079, China

**Keywords:** DYRK1A, heterocyclic inhibitors, classification models, pharmacophoric fragments

## Abstract

Dual-specific tyrosine phosphorylation regulated kinase 1 (DYRK1A) has been regarded as a potential therapeutic target of neurodegenerative diseases, and considerable progress has been made in the discovery of DYRK1A inhibitors. Identification of pharmacophoric fragments provides valuable information for structure- and fragment-based design of potent and selective DYRK1A inhibitors. In this study, seven machine learning methods along with five molecular fingerprints were employed to develop qualitative classification models of DYRK1A inhibitors, which were evaluated by cross-validation, test set, and external validation set with four performance indicators of predictive classification accuracy (CA), the area under receiver operating characteristic (AUC), Matthews correlation coefficient (MCC), and balanced accuracy (BA). The PubChem fingerprint-support vector machine model (CA = 0.909, AUC = 0.933, MCC = 0.717, BA = 0.855) and PubChem fingerprint along with the artificial neural model (CA = 0.862, AUC = 0.911, MCC = 0.705, BA = 0.870) were considered as the optimal modes for training set and test set, respectively. A hybrid data balancing method SMOTETL, a combination of synthetic minority over-sampling technique (SMOTE) and Tomek link (TL) algorithms, was applied to explore the impact of balanced learning on the performance of models. Based on the frequency analysis and information gain, pharmacophoric fragments related to DYRK1A inhibition were also identified. All the results will provide theoretical supports and clues for the screening and design of novel DYRK1A inhibitors.

## 1. Introduction

Protein kinases are implicated in cellular functions by transferring a chemical addition of phosphate group to proteins [1]. Dual-specific tyrosine phosphorylation regulated kinases (DYRKs), which include class I (DYRK1A and DYRK1B) and class II (DYRK2, DYRK3 and DYRK4) possess a dual specificity capability to phosphorylate tyrosine residue Y321 in its own activation loop as well as to phosphorylate its substrates at serine or threonine residues [2]. Among the mammalian DYRKs, DYRK1A is involved in the proliferation and differentiation of the central nervous system (CNS) ranging from early embryogenesis to late aging. Consequently, DYRK1A has been considered as a potential therapeutic target of neurodegenerative diseases such as Alzheimer’s disease (AD) and Down’s syndrome (DS) [3]. Inhibition of DYRK1A attenuates cognitive dysfunctions in animal models for both AD and DS disease [4]. 

At present, a diversity of DYRK1A inhibitors were identified as potential therapies for neurodegenerative diseases including natural product harmine and synthetic compounds, benzothiazoles, azaindoles, pyrazolo, pyridine, pyrimidine, and quinazolines [5,6]. However, due to the closely relationship of DYRK1A with CMGC family kinases, most reported inhibitors are impeded from clinical trials because of lower selectivity and other pharmaceutics deficiencies [7,8]. Therefore, considerable attempts are focused on the discovery of novel potent and selective DYRK1A inhibitors, which has been accelerated through the method of structure and fragment-based drug design [9]. For example, imidazopyridine [10] and pyrrolopyrimidine compounds [11] were identified as potent and selective DYRK1A inhibitors using fragment-based hit discovery and structure-based optimization strategy. Harmine derivatives were also optimized as potent GSK-3β/DYRK1A dual inhibitors for the treatment of Alzheimer’s disease [12]. Binding modes and pharmacophoric features are regarded as two prerequisite conditions for structure and fragment-based drug design. X-ray crystallography and molecular modeling studies provide the detailed binding modes of DYRK1A-inhibtor complexes. DYRK1A has the conventional fold of a protein kinase with N- and the C-terminal lobes, forming a hydrophobic deep cleft where the co-substrate can bind. The proper orientations of ATP and catalytic residues are crucial for catalysis. Specifically, the chemical scaffold of ATP is sandwiched into a hydrophobic pocket (Val173, Phe238, Leu241, Leu294, Val306, and Phe308). Meanwhile the adenine moiety and phosphates are anchored by two hydrophilic regions. In particular, a so-called hinge region mainly including resides Glu239, Met240, and Leu241 forms a couple of hydrogen bonds with the adenosine moiety. A segment known as positive electrostatic area (positive area) involving Lys188, Glu203, and Asp307 contributes to the stabilization of α- and β-phosphates of ATP [11,13]. As for the discovery of pharmacophoric fragments, biophysical techniques are powerful tools of fragment screening, but necessitate expensive detection equipment with time-consuming and low-hit rate. Meanwhile, computational and chemical informatics chemistry methods including quantitative structure–activity relationship (QSAR) and data mining tools can be used as complementary approaches to identify structural features and properties of inhibitors that are strictly connected with their biological activities [14]. Bharate et al. [15] developed a descriptor based QSAR model of Meridian derivatives and identified crucial molecular descriptors for their biological activity. Hologram QSAR (2D fragment-based) models of 6-arylquinazolin-4-amine inhibitors indicated the fragments that made positive contributions to their biological activity [16]. However, the above-mentioned QSAR studies were based on the compounds with one certain scaffold, and thus only provided exclusive optimization clues for the specific scaffold. Developing a QSAR model involving DYRK1A inhibitors with diverse chemical scaffolds could provide general and comprehensive molecular information or privileged substructures that are determinative factors to their inhibitory activity.

Recently, machine learning (ML) methods have been considered as powerful tools to build robust and predictive classification models [17,18]. Without the limitation of data samples in one certain chemical scaffold, classification studies of machine learning methods along with molecular features [19,20] are applicable for DYRK1A inhibitors with diverse heterocyclic scaffolds and broad-spectrum bioactivities. Here, classification models of DYRK1A inhibitors were developed using seven machine learning methods combined with five molecular fingerprints. Furthermore, a hybrid data balancing method SMOTETL, a combination of the synthetic minority over-sampling technique (SMOTE) and Tomek link (TL) algorithms, was applied to explore the prediction capacity of imbalanced learning to unbalanced data. All models were further evaluated by five-fold cross validation method, a test set, and external validation set. A combination of frequency analysis and information gain was also performed to identify the pharmacophoric fragments related to DYRK1A inhibition. All the results provide comprehensive structural clues for the discovery of DYRK1A inhibitors.

## 2. Results and Discussion

### 2.1. Dataset Analysis

**Figure 1:**
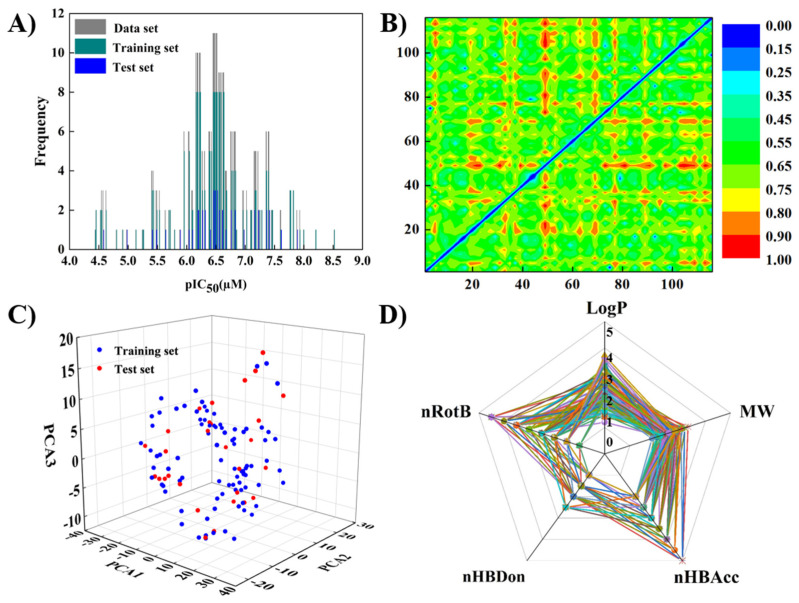
(**A**) Distributions of experimental pIC_50_ values for the dataset ( 117, grey bars), training set (88, green bars), and test set (29, blue bars). (**B**) Heat map of the molecular similarity constructed by Euclidian distance metrics for the entire dataset. (**C**) Chemical space of the training set (blue dots) and test set (red dots) using top three principal components of dragon molecular descriptors (51% variance explained). (**D**) Radar map of the dataset with the parameters of Lipinski’s rules of five and the number of rotatable bonds.

As shown in Figure 1A, the experimental pIC_50_ values of the whole dataset ranged from 4.5 to 8.5 and were mainly distributed around 6.0 and 7.0. As 6.0 is the approximate average value of the pIC_50_ values for all datasets, pIC_50_ = 6.0 (IC_50_ = 1 μM) was considered as a threshold to define the potent and non-potent inhibitors. Based on a cut-off value IC_50_ = 1 μM, this dataset was split into 89 potent inhibitors (“P”) and 28 non-potent (“N”), among which 88 training set compounds (69 potent inhibitors and 19 non-potent inhibitors with of the ratio of 3.5:1) and 29 test set compounds (20 potent inhibitors and nine non-potent inhibitors with the ratio of 2:1), respectively. Given the roughly balanced distribution of “P” inhibitors (training set = 78.4%, test set = 68.9%), each group was suitable to evaluate the predictive performance of the models.

Chemical diversity is an important index of similarity among molecules to build a robust and predictable model. A heat map constructed by Euclidian distance metrics (calculated by PubChem fingerprint) was used to characterize the chemical diversity of molecules. As indicated from Figure 1B, red (1) and blue (0) illustrated the highest and lowest diversity of molecules, respectively. Most plots were distributed in the green area (around 0.4), which indicated that the dataset presented high diversity, and the models trained based on such data can have strong generalization ability. Chemical spaces of the whole dataset were investigated based on PCA analysis of featured molecular descriptors, four descriptors of Lipinski rules, and the number of rotatable bonds. Since 51% featured descriptor variance of this dataset was explained by the top three most principal components, the training set and test could be regarded as in the similar chemical spaces (Figure 1C). Additionally, there is no remarkable preference of four descriptors of Lipinski’s rules of five and the number of rotatable bonds of the entire dataset (Figure 1D). Consequently, in light of the chemical spaces and structural diversity of the whole dataset, the basic requirements of a reliable classification model were qualified.

### 2.2. Five-Fold Cross Validation Results

**Figure 2:**
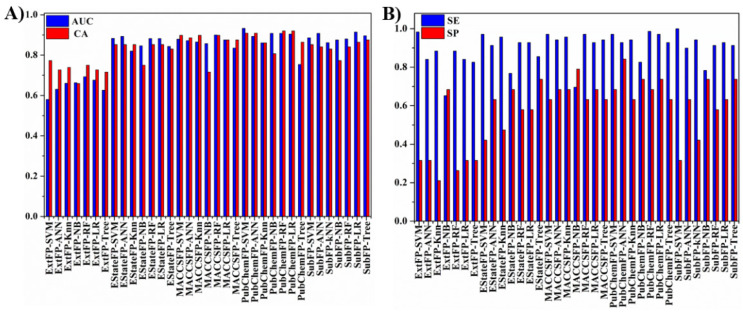
Performance of the training set with five-fold cross validation of 35 models. (**A**) AUC-CA histogram; (**B**) SE-SP histogram.

A 5-fold cross-validation for the training set was performed to evaluate the performance of all developed models. The top ten ranking modes were screened by taking overall predictive classification accuracy (CA) and the area under the ROC curve (AUC) as indicators. As shown in Figure 2A, all the models yielded CA and AUC values higher than 0.6. Interestingly, regardless of which ML method was employed, Ext fingerprint yielded the lowest CA and AUC values under 0.8, which were smaller than those of the other models with CA and AUC values (ranging from 0.8 to 0.9). Meanwhile, in light of the higher predictive accuracy of “P” class (sensitivity, SE) than “N” class (specificity, SP) values shown in Figure 2B, it can be speculated that all modes exhibited a better predictive ability for “P” inhibitors than “N” inhibitors. This may be due to the unbalancing “P” inhibitors in the training set with a ratio of 0.78.

Table 1 lists the detailed performances of ten models. Both PubChem and Sub fingerprints generated the optimal results in general, especially five modes derived from PubChem fingerprint combined with any ML algorithm. Taking the CA, AUC, BA (average of SE and SP), and Matthews’s correlation coefficient (MCC) as indicators, PubChem-SVM and PubChemFP-ANN were the top two models with high predictive ability.

### 2.3. Performance of the Test Set

The predictive ability of ten models was further evaluated by a test set. Similar to those of the five-fold cross-validation results, AUC and CA values were in the range of 0.8 to 0.9 in ten models for the test set, whereas the SE and SP values exhibited different behaviors such as the higher SE than SP in Sub-, MACCS-, and Estate-based and PubChem-RF models, and similar values of SE and SP (0.800 or 0.850 and 0.899, respectively) in four PubChem-based models. Consequently, in light of the higher CA, AUC, MCC, and BA values, PubChem-SVM and PubChem-ANN yielded good performances for the test set.

### 2.4. Predicted Results of External Validation Set

Although several models based on the PubChem and Sub fingerprint generated good performance for the training and test set, these models were still needed to be further evaluated by an external validation set. The external validation set contained different types of compounds collected from the relevant literature including benzofuranyl, indole, pyrrolidinyl, and carbazolyl derivatives, which were not included in the training and test set. The predicted results of the external validation set are shown in Table 2. In light of the AUC, CA, and MCC values as the performance indicators, the Sub-ANN and PubChem-ANN models gave the top three predictive results for the external validation set in general. It could also be found that all models obtained higher SE values than SP values, which reflected that these models generated better predictive ability for “P” compounds of the external validation set.

### 2.5. Improved Performance of Balanced Models

Given the high degree of imbalance of the datasets, a hybrid balancing method SMOETL was applied to explore the impact of balanced learning on the performance of models. Here, the comparisons between balanced and imbalanced modes are discussed. As indicated in Table 2, the performances of balanced models were remarkably improved in contrast to those of the imbalanced models for the training set, especially the similar values of SE and SP and the approximate BA values of 0.95, which implied that these models have predictive capacity for both “P” and “N” samples. For the test set, except for the higher SE than SP occurring in all balanced modes (vs. the similar SE and SP of four imbalanced models), there were no observable changes (AUC, CA, MCC, and BA values) between the imbalanced and balanced models. All the balanced models were sensitive to predict potent compounds for the test set. Regarding the external validation set, balanced models such as MACCSFP-LR and Sub-LR were the most predictive models for the external validation set. Compared to the statistical parameters of the imbalanced models, the reduced gap between SE and SP values occurred in the balanced models, which meant that the SMOTETL balancing method indeed enhanced the performances of models for the external validation test. However, since four compounds (123, 125, 126, and 131) in the external validation set possessed a unique chemical structure with four heterocyclic rings connected by fusing or double bonds, which were different to the structural features of the training set composed of three heterocyclic rings with a single bond topological connection, the classifier developed based on the training set generated a weaker performance on the external set. Meanwhile, due to the smaller sample in the external validation set (10 “P” and 5 “N”), balanced learning only generated a slight improvement in the prediction capacity. Interestingly, in light of the high ratio of positive compounds in the external validation set, which shared the similar chemical scaffolds with training set, both imbalanced and balanced models were applicable to predict the “P” class.

Based on the performances of models for the training set, test set, and external test set, thee PubChem fingerprint was involved in the best model for the entire dataset. ANN and SVM were considered as optimal methods for establishing the classification prediction model of DYRK1A heterocyclic inhibitors. As PubChem fingerprint (881 bits) is a well-defined structural fragment dictionary with a variety of different substructures and features, the chemical fragments responsible for DYRK1A inhibition will be analyzed based on the PubChem fingerprint.

### 2.6. Identification and Analysis of Feature Substructures

In order to identify chemical fragments responsible for DYRK1A inhibition, IG and frequency analysis were performed to screen the feature substructures based on the PubChem fingerprint. The higher the IG values, the greater contribution of the feature substructures to DYRK1A inhibition. Sixteen positive and 10 negative fingerprints that occur more frequently in “P” inhibitors and “N” inhibitors responsible for DYR1KA modulation/inhibition are presented in Table S4. Since positive fingerprints could be used as the structural signs to discover and screen novel potent DYRK1A inhibitors, representative substructures (Table 3) presented in “P” class with high ratios were focused on and discussed in detail.

**Figure 3:**
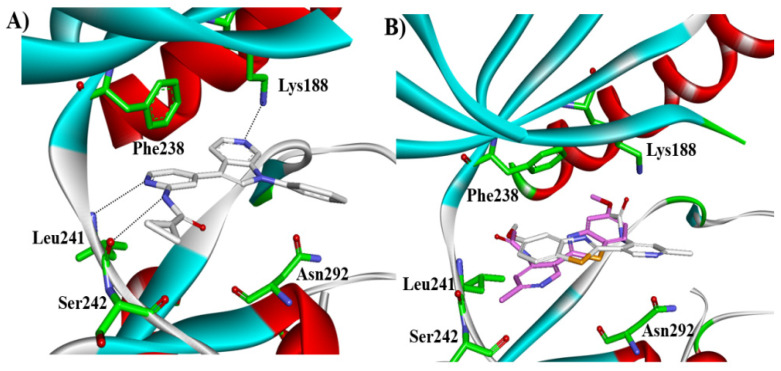
Proposed binding modes of (**A**) compound 73 and (**B**) compound 23 with DYRK1A.

Most DYRK1A inhibitors possessed a heterocyclic scaffold corresponding to the privileged fragment of ≥3 hetero-aromatic rings (PubChemFP260), which was strongly associated with the protein by hydrophobic interactions with Ile165, Val173, Ala186, Leu241, Leu294, and Val306 side chains. Furthermore, the hetero-aromatic pyridine (PubchemFP187 and PubchemFP188) was also involved to produce a hydrogen bond with a backbone NH of Leu241, and a pyridyl nitrogen of 6-azaindole (PubchemFP499, PubchemFP547, PubchemFP569, and PubchemFP611) forms electrostatic interactions with the side chain NH_2_ group of Lys188 in the positive area. Therefore, it is reasonable to understand the potency of 6-azaindole derivatives against DYRK1A with IC_50_ values ranging from 0.0062 to 0.315. Another remarkable privileged substructure, 2-hydroxy or 2-alkoxyl benzothiazole (PubchemFP691, PubchemFP702 and PubchemFP703, or PubchemFP720 and PubchemFP783) was found in compounds 1 (IC_50_ = 0.056 μM) and 24 (IC_50_ = 0.0938 μM), in which the hydoxyl (alkoxyl) group formed two hydrogen bonds (single H-bond) with Leu241 in the hinge region. Additionally, an alternative binding mode was also found for 2-alkoxyl compounds that flipped and switched the binding interaction from the hinge region (Leu241) to the positive area (Lys188). By comparing the structures and activities of compounds 46 (IC_50_ = 28.1 μM) and 47 (IC_50_ = 0.8 μM), an alkoxyl at R2 generated a 35-fold increased inhibitory activity in contrast to an alkoxyl at the R3 position. Acetamide groups (PubChemFP645 and PubChem646) were also identified as a pharmacophoric group since it produced polar interactions either with the hinge region or the positive area, depending on the chemical scaffolds substituted on. For example, compound 73 (IC_50_ = 0.301 μM) with the acetamide amide made an additional hydrogen bond with backbone carbonyl oxygen atoms of Leu241 (Figure 3A). However, the acetamide carbonyl substituted on pyridine of compound 23 formed polar interactions with the side chain NH_2_ group of Lys188 or the backbone NH of Leu241 in the positive area alternatively (Figure 3B). Furthermore, the additional pyridine linked to benzothiazole might be helpful to form the π–interaction with the phenyl of Phe238, which provides a structural basis for the improved potency of compound 23 (IC_50_ = 0.15 μM) in contrast to compound 53 (IC_50_ = 3.9 μM) with acetamide.

Fragment-based drug design including fragment-based growing and/or linking strategies and fragment hybridization strategies has been widely used in drug discovery by assembling fragments into novel molecules. Here, the privileged substructures above-mentioned provide structural elements for the discovery of DYRK1A inhibitors. AlNajjar et al. [21] reported that 6-hydroxybenzothiazole urea derivatives b27 displayed the highest potency against DYRK1A with an IC_50_ of 20 nM, which could be considered as an acetamide group-based linking compound by connecting 6-hydroxybenzothiazole and a phenyl acetamide together. Docking results indicated that hydrogen bonds were formed between b27 and key residues Leu241, Glu239, and Lys188 and/or Asp307 in the active site of DYRK1A including acetamide groups with the hinge region backbone of Leu241 and Glu239 as well as between the OH of 6-hydroxybenzothiazole and the conserved Lys188 and/or Asp307. Meanwhile, the above-mentioned substructures such as pyridine and hydroxyl-phenyl ring were also presented in 1H-pyrazolo [3,4-b]pyridine derivatives 8 h (IC_50_ = 5 nM) [22]. Among them, the 1H-pyrazolo[3,4-b]pyridine scaffold were involved in the interactions with the Glu239 backbone CO and Leu241 backbone NH in the hinge region and the phenol ring formed electrostatic interaction with Lys188.

**Figure 4:**
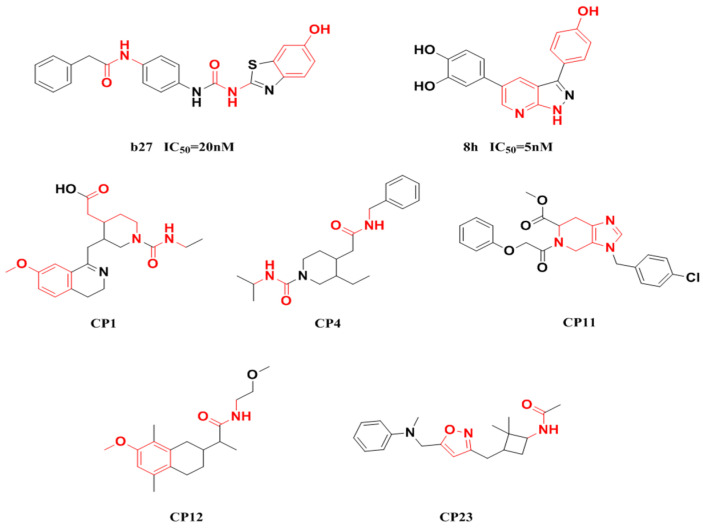
Pharmacophoric fragments presented in the DYRK1A inhibitors (b27 and 8 h) and theoretical hits.

**Figure 5:**
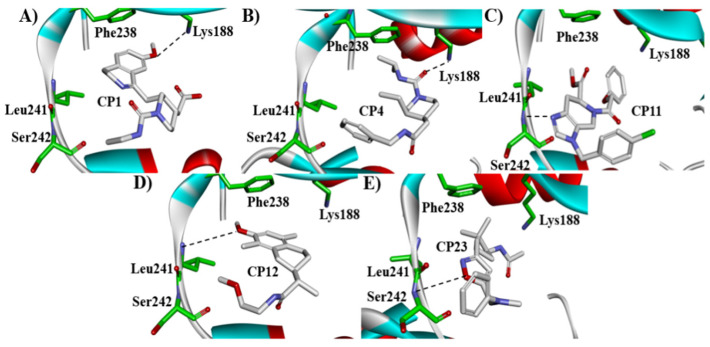
Predicted binding modes of five theoretical hits, (**A**) CP1, (**B**) CP4, (**C**) CP11, (**D**) CP12 and (**E**) CP23 with DYRK1A.

Besides the validation of classification modes by the reported inhibitors, a hybrid virtual screening of natural products including pharmacophore hypothesis, classification models, and molecular docking was also performed to identity DYRK1A inhibitors with new scaffolds [undergoing work]. As shown in Figure 4, five compounds (CP1, CP4, CP11, CP12, and CP23) were screened as the top five theoretical hits, which will be further evaluated and tested by the kinase assay. Interestingly, five hits possessed the privileged fragments derived from classification models, which were predicted to produce polar interactions with the hinge region and/or positive area as the above-mentioned inhibitors form (Figure 5). For instance, 2-benzyloxy of CP1 and CP12 may have polar interactions with a side chain NH_2_ group with a Lys188 and Leu241 backbone NH, respectively. It is possible for the acetamide group of CP4 to interact with Lys188. Imidazole (CP111) and isoxazole (CP23) are also expected to form hydrogen bonds with Leu241 and/or Ser242 of the hinge region.

## 3. Material and Methods

### 3.1. Data Collection and Chemical Diversity

By considering the diversity of the chemical scaffold and the distribution of inhibitory activity, 117 DYRK1A heterocyclic inhibitors were used as the whole dataset [23,24,25,26,27,28,29], which were divided into a training set and a test set with a ratio of 3:1 (Table S1). In addition, an external validation set including 15 compounds (10 “P” and 5 “N”, Table S2) was also used to further evaluate the robustness and reliability of classification models. The detailed information of whole dataset was listed in Table 4.

A heat map of the Euclidian distance metrics based on PubChem fingerprints was employed as an indicator of molecular similarity of compounds. In order to evaluate chemical space covered by the entire dataset, 825 2D molecular descriptor groups (e.g., constitutional indices, charge descriptors, ring descriptors, topological indices, connectivity indices, etc.) were calculated by DRAGON 7.0 [30]. In order to avoid the over-fitting possibility of these descriptors, a preliminary screening was applied to exclude the constant and nearly constant variance (>80% compounds sharing the same values with a descriptor) and descriptors with high inter-correlation (pair-wise correlations among all pairs of descriptors >95%). Then, 634 descriptors were further evaluated by principal component analysis to identify the featured molecular descriptors. The descriptors of Lipinski’s rules of five [31] were also plotted into a radar chart to observe the chemical space distribution of the entire dataset.

### 3.2. Molecular Fingerprints and Machine Learning Methods

Molecular fingerprint belongs to a kind of chemical structure feature, which is widely used in similarity search, clustering, or recursive partition [32]. The two-dimensional or three-dimensional characteristics of molecules are compiled into binary values (0 for none, 1 for have) or counts, and the chemical structure is converted into a data format that can be understood by a computer. In this study, five molecular fingerprints namely Molecular ACCess System (MACCS, 166 bits), Extended (Ext, 79 bits), Estate (Est, 1024 bits), PubChem (881 bits), and Substructure (Sub, 307 bits) were calculated using PaDEL-Descriptors software [31].

In this study, the classification models were built using the support vector machine (SVM), logistic regression (LR), k-nearest neighbor (KNN), artificial neural network (ANN), naïve Bayes (NB), random forest (RF) (with a tree number of 20 and the maximum tree depth of 15), and decision tree (DT). An in-depth description of the application of these methods in drug discovery can be obtained from some excellent studies and research papers [33,34]. All of these calculations were integrated with Orange Canvas 3.11 software (freely available at https://orange.biolab.si/, accessed on 8 March 2018).

In order to explore the impact of balanced learning on the model’s performance, SMOTETL was applied on the original training set to balance the number of potent and non-potent samples. The test set and external validation set were kept unbalanced. This hybrid technique combines oversampling and under sampling techniques. The Tomek link consists of two samples that are the nearest neighbor but do not belong to the same class. This under sampling technique eliminates the observations of the majority class. The SMOTE technique oversamples the original dataset and then detects and removes those samples that compose the Tomek link [35].

### 3.3. Model Performance Evaluation

In order to obtain a comprehensive evaluation of models, the five-fold cross validation method, a test set and an external test set were employed to evaluate the developed classification models based on statistical parameters including *TP*, *TN*, *FP*, *FN*, SE, SP, CA, and balanced accuracy (average of SE and SP) [36]. MCC was also explored to measure the correlation between the true class labels and the predicted labels.

(1) $$\mathrm{SE}=\frac{TP}{TP+FN}$$

(2) $$\mathrm{SP}=\frac{TN}{TN+FP}$$

(3) $$\mathrm{CA}=\frac{TP+TN}{TP+TN+FP+FN}$$

(4) $$\mathrm{MCC}=\frac{TP\times TN-FP\times FN}{\sqrt{\left(TN+FN\right)\left(TN+FP\right)\left(TP+FN\right)\left(TP+FP\right)}}$$

In addition, the receiver operating characteristic (ROC) curve was also plotted based on the TP and FP rates. AUC ranging from 0.5 to 1.0 was also used to evaluate the accuracy performance of the classification models. If the AUC value is 1.0, it is considered as a perfect classifier. If the AUC is 0.5, it is considered as a classifier without discriminative ability [37].

(5) $$\mathrm{AUC}=\int_{t=\infty }^{-\infty }y\left(t\right)dx_{t}$$

### 3.4. Identification of Privileged Substructures

Information gain value (IG) and substructure frequency contribution were explored to identify privileged groups of DYRK1A inhibitors. If a fragment appears more frequently in the “P” class, this fragment is regarded as a privileged substructure of the DYRK1A inhibitor. The formula is defined as follows:

(6) $$\mathrm{Frequency}\;\mathrm{of}\;a\;\mathrm{substructure}=\frac{N_{fragment}^{I}\times N_{total}}{N_{fragment-total}\times N_{I}}$$

where $N_{fragment}^{I}$ is the number of compounds in the “P” class containing a fragment$;\;N_{total}$ is the number of inhibitors in the entire dataset; $N_{fragment-total}$ is the number of inhibitors in the entire dataset containing a certain fragment; and $N_{I}$ is the total number of “P” inhibitors in the whole dataset.

### 3.5. Molecular Docking

In order to predict the binding modes of theoretical hits with DYRK1A, molecular docking was performed by using AutoDock Vina v1.2.0 [38]. The active site of the receptor was defined as a three-dimensional grid of (50 × 50 × 50) points with a grid spacing of 0.375Å at the center of mass of the ligand (PDB ID: 3ANR) [39]. The Lamarckian genetic algorithm (LGA) was employed as the conformational search method to explore the binding modes between DYRK1A and the inhibitors.

## 4. Conclusions

In this study, classification studies of 117 DYRK1A inhibitors were explored using machine learning methods along with molecular fingerprints. Based on the performances of models evaluated by 5-fold cross validation and the test set, the PubChem fingerprint was involved in the best model for the training set and test set with an accuracy of 0.933 and 0.911 when combined with the SVM and ANN algorithm, respectively. Furthermore, pharmacophoric substructures related to their inhibitory activity were also identified using information gain and substructure frequency analysis. All these results provide the theoretical basis to understand key groups responsible for DYRK1A inhibition as well as the valuable hints for the discovery of novel DYRK1A inhibitors.

Recently, ML algorithms have widely been used in fragment-based drug discovery. The statistical ML models are able to develop the categorical or continuous correlations between molecular features and compound activity/property and make predictions for new chemical entities. In particular, the categorical correlation derived from classification models offers an attractive approach for exploring the pharmacophoric fragments to bind a target protein. Continuous correlations of QSAR models can be used to filter the fragment-based optimization compounds with the desired activities and properties in silicon. In light of the limited learning capability of ML algorithms, the developed models may be insufficient to generalize well across different structures and identify the exclusive functional groups with over-dependence on the training set. However, the developed models exhibited excellent prediction ability to the compounds that shared similar molecular information to the training set, and also used to identify the novel DYRK1A inhibitors from the natural product dataset. In the future, with the increase in storage capacity and the size of the dataset available, a subfield of ML called deep learning (DL)-based models will be applicable in drug discovery by means of not only learning from a dataset but also generating new data in a multidimensional way. Therefore, DL-derived models generalize well across compounds with more diverse chemical scaffolds and produce an entire prediction in more broad application domains.

## Figures and Tables

**Table 1:** Performance of top 10 models for the training set, test set, and external validation set.

Data Set	Model	AUC	CA	MCC	TP *	TN *	FP *	FN *	SE	SP	BA
Training set	PubChemFP-SVM	0.933	0.909	0.717	67	13	6	2	0.971	0.684	0.828
	SubFP-LR	0.914	0.864	0.583	64	12	7	5	0.928	0.632	0.780
	PubChemFP-NB	0.908	0.807	0.508	57	14	5	12	0.826	0.737	0.782
	PubChemFP-RF	0.908	0.920	0.753	68	13	6	1	0.986	0.684	0.835
	SubFP-ANN	0.908	0.841	0.530	62	12	7	7	0.899	0.632	0.766
	PubChemFP-LR	0.904	0.920	0.755	67	14	5	2	0.971	0.737	0.854
	MACCSFP-RF	0.900	0.898	0.678	67	12	7	2	0.971	0.632	0.802
	SubFP-Tree	0.896	0.875	0.638	63	14	5	6	0.913	0.737	0.825
	EStateFP-ANN	0.893	0.852	0.556	63	12	7	6	0.913	0.632	0.773
	PubChemFP-ANN	0.893	0.909	0.743	64	16	3	5	0.928	0.842	0.885
Test set	PubChemFP-SVM	0.911	0.862	0.705	17	8	1	3	0.850	0.889	0.870
	SubFP-LR	0.903	0.793	0.493	18	5	4	2	0.900	0.556	0.728
	PubChemFP-NB	0.881	0.828	0.647	16	8	1	4	0.800	0.889	0.845
	PubChemFP-RF	0.917	0.897	0.761	20	6	3	0	1.000	0.667	0.834
	SubFP-ANN	0.881	0.793	0.517	17	6	3	3	0.850	0.667	0.759
	PubChemFP-LR	0.944	0.862	0.705	17	8	1	3	0.850	0.889	0.870
	MACCSFP-RF	0.922	0.862	0.680	20	5	4	0	1.000	0.556	0.778
	SubFP-Tree	0.825	0.862	0.517	17	6	3	3	0.850	0.667	0.759
	EStateFP-ANN	0.858	0.793	0.517	17	6	3	3	0.850	0.667	0.759
	PubChemFP-ANN	0.911	0.862	0.705	17	8	1	3	0.850	0.889	0.870
Validation set	PubChemFP-SVM	0.660	0.667	0.213	8	2	3	2	0.800	0.400	0.600
	SubFP-LR	0.780	0.667	0.139	9	1	4	1	0.900	0.200	0.550
	PubChemFP-NB	0.660	0.667	0.378	6	4	1	4	0.600	0.400	0.500
	PubChemFP-RF	0.430	0.600	−0.189	9	0	5	1	0.600	0.000	0.300
	SubFP-ANN	0.760	0.733	0.354	9	2	3	1	0.900	0.400	0.650
	PubChemFP-LR	0.760	0.667	0.139	9	1	4	1	0.600	0.100	0.350
	MACCSFP-RF	0.820	0.667	-	10	0	5	0	1.000	0.000	0.500
	SubFP-Tree	0.600	0.667	0.213	8	2	3	2	0.800	0.400	0.600
	EStateFP-ANN	0.820	0.667	0.213	8	2	3	2	0.800	0.400	0.600
	PubChemFP-ANN	0.660	0.733	0.354	9	2	3	1	0.900	0.400	0.650

* True positive (TP), true negative (TN), false positive (FP), and false negative (FN).

**Table 2:** Performance of top 10 balanced models for the training set, test set, and external validation set.

Data Set	Model	AUC	CA	MCC	*TP*	*TN*	*FP*	*FN*	SE	SP	BA
Training set	PubChemFP-LR	0.993	0.948	0.896	63	64	3	4	0.940	0.955	0.948
	PubChemFP-SVM	0.990	0.940	0.881	64	62	5	3	0.955	0.925	0.940
	PubChemFP-ANN	0.989	0.955	0.910	64	64	3	3	0.955	0.955	0.955
	MACCSFP-RF	0.984	0.954	0.908	62	62	3	3	0.954	0.954	0.954
	PubChemFP-kNN	0.983	0.948	0.896	62	65	2	5	0.925	0.970	0.948
	PubChemFP-RF	0.979	0.948	0.896	65	62	5	2	0.970	0.925	0.948
	MACCSFP-LR	0.974	0.954	0.908	62	62	3	3	0.954	0.954	0.954
	MACCSFP-kNN	0.972	0.954	0.908	61	63	2	4	0.938	0.969	0.954
	SubFP-RF	0.971	0.888	0.777	61	58	9	6	0.910	0.866	0.888
	SubFP-LR	0.971	0.881	0.761	59	59	8	8	0.881	0.881	0.881
Test set	PubChemFP-LR	0.808	0.863	0.577	19	5	4	1	0.950	0.556	0.753
	PubChemFP-SVM	0.883	0.828	0.680	20	5	4	0	1.000	0.556	0.778
	PubChemFP-ANN	0.836	0.828	0.493	18	5	4	2	0.900	0.556	0.728
	MACCSFP-RF	0.933	0.862	0.667	19	6	3	1	0.950	0.667	0.808
	PubChemFP-kNN	0.881	0.862	0.680	20	5	4	0	1.000	0.556	0.778
	PubChemFP-RF	0.895	0.862	0.697	20	7	3	1	0.952	0.700	0.826
	MACCSFP-LR	0.922	0.862	0.667	19	6	3	1	0.950	0.667	0.808
	MACCSFP-kNN	0.811	0.862	0.680	20	5	4	0	1.000	0.556	0.778
	SubFP-RF	0.872	0.862	0.680	20	5	4	0	1.000	0.556	0.778
	SubFP-LR	0.806	0.759	0.393	18	4	5	2	0.900	0.444	0.672
Validation set	PubChemFP-LR	0.500	0.600	0.100	7	2	3	3	0.700	0.400	0.550
	PubChemFP-SVM	0.400	0.600	0.100	7	2	3	3	0.700	0.400	0.550
	PubChemFP-ANN	0.560	0.533	0.000	6	2	3	4	0.600	0.400	0.500
	MACCSFP-RF	0.640	0.667	0.139	9	1	4	1	0.900	0.200	0.550
	PubChemFP-kNN	0.330	0.600	−0.189	9	0	5	1	0.900	0.000	0.450
	PubChemFP-RF	0.300	0.600	−0.189	9	0	5	1	0.900	0.000	0.450
	MACCSFP-LR	0.760	0.733	0.378	10	1	4	0	1.000	0.200	0.600
	MACCSFP-kNN	0.590	0.533	−0.277	8	0	5	2	0.800	0.000	0.400
	SubFP-RF	0.760	0.773	0.378	10	1	4	0	1.000	0.200	0.600
	SubFP-LR	0.520	0.600	−0.189	9	0	5	1	0.900	0.000	0.450

**Table 3:** PubChem fingerprint-based privileged substructures responsible for DYR1KA inhibition.

Fingerprints	Substructure	General Substructure	Representative Substructure	IG	FP	FN
PubchemFP187	≥2 saturated or aromatic nitrogen-containing ring size 6	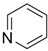	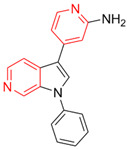	0.088	1.315 (23)	0 (0)
PubchemFP188	≥2 saturated or aromatic heteroatom-containing ring size 6			0.088	1.315 (23)	0 (0)
PubchemFP260	≥3 hetero-aromatic rings	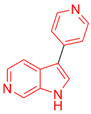	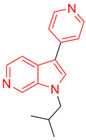	0.067	1.292 (18)	0 (0)
PubchemFP646	O=C–N–C–[#1]	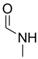	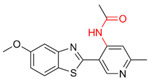	0.063	1.315 (17)	0 (0)
PubchemFP645	O=C–N–C–C		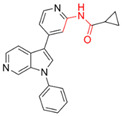	0.053	1.230 (29)	0.270 (2)
PubchemFP499	N–C:C:N	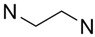	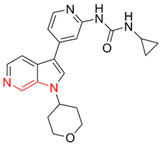	0.064	1.237 (32)	0.246 (2)
PubchemFP547	N–C:C-N			0.064	1.237 (32)	0.246 (2)
PubchemFP569	N–C–C–N			0.060	1.213 (36)	0.321 (3)
PubchemFP611	N–C–C–N–C			0.060	1.213 (36)	0.321 (3)
PubchemFP629	S-C:C:C-N	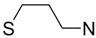	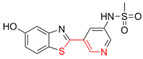	0.062	1.198 (41)	0.371 (4)
PubchemFP658	C–C–S–C–C	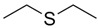	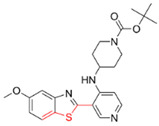	0.062	1.198 (41)	0.371 (4)
PubchemFP691	O–C–C–C–C–C–N	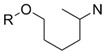	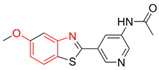	0.144	1.263 (49)	0.164 (2)
PubchemFP702	O–C–C–C–C–C–N–C			0.144	1.263 (49)	0.164 (2)
PubchemFP703	O–C–C–C–C–C(N)–C			0.139	1.262 (48)	0.167 (2)
PubchemFP720	Oc1ccc(S)cc1	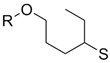	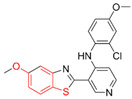	0.103	1.253 (41)	0.194 (2)
PubchemFP783	OC1CCC(S)CC1			0.103	1.253 (41)	0.194 (2)

**Table 4:** The statistics of molecules in the datasets.

Data Set	Potent Inhibitors (P)	Non-Potent Inhibitors (N)	Total
Train set	69	19	88
Test set	20	9	29
Validation set	10	5	15
Total	99	33	132

## Supplementary Materials

The following supporting information can be downloaded at: https://www.mdpi.com/article/10.3390/molecules27061753/s1, Table S1: Structure and inhibitory activity of DYRK1A inhibitors of the training and test set; Table S2: Structure and inhibitory activity of DYRK1A inhibitors of the external validation set; Table S3: Performance of 35 classification models for the training set and test set; Table S4 PubChem fingerprints of inhibitors (16) and non-inhibitors (10) responsible for DYR1KA modulation/inhibition.

## Sample Availability

Samples of the compounds are available from the authors.
