# Supplementary material for: Identification of Pharmacophoric Fragments of DYRK1A Inhibitors Using Machine Learning Classification Models
Source: Molecules. 2022 Mar 8;27(6):1753. doi: 10.3390/molecules27061753 (PMC8954712; doi:10.3390/molecules27061753)
Supplement: Supplementary file 1 [file molecules-27-01753-s001.zip › molecules-1632138-supplementary.pdf]

Identification of Pharmacophoric Fragments of DYRK1A Inhibitors using Machine Learning Classification Models

Mengzhou Bi [1,†], Zhen Guan [2,†], Tengjiao Fan [1,3], Na Zhang [1,*], Jianhua Wang [2,*], Guohui Sun [1], Lijiao Zhao [1] and Rugang Zhong [1]

[1] Key Laboratory of Environmental and Viral Oncology, College of Life Science and Chemistry, Faculty of Environment and Life, Beijing University of Technology, Beijing 100124, China; bimengzhou@emails.bjut.edu.cn (M.B.); fannie818@126.com (T.F.); sunguohui@bjut.edu.cn (G.S.); zhaolijiao@bjut.edu.cn (L.Z.); lifesci@bjut.edu.cn (R.Z.)
[2] Beijing Municipal Key Laboratory of Child Development and Nutriomics, Translational Medicine Laboratory, Capital Institute of Pediatrics, Beijing 100020, China; mengai518@126.com
[3] Department of Medical Technology, Beijing Pharmaceutical University of Staff and Workers, Beijing 100079, China
* Correspondence: nanatonglei@bjut.edu.cn (N.Z.); wangjianhua@shouer.com.cn(J.W.)
† These authors contributed equally to this paper.

Table S1. Structure and activity of DYRK1A inhibitors of the training and test set.

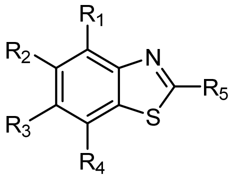

1–64

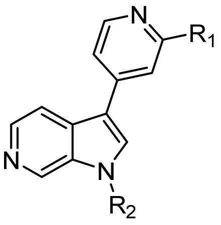

65–80

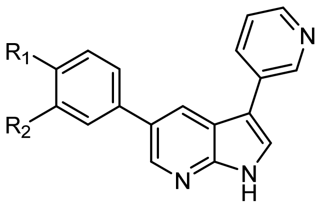

81–82

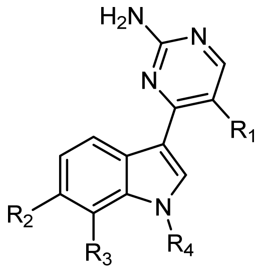

83–91

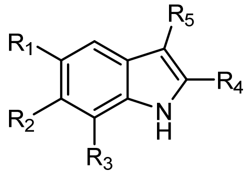

92–117

No.	R1	R2	R3	R4	R5	IC50 (μM)	P/N
1	H	-OCH3	H	H	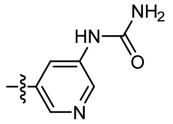	0.056	P
2*	H	-OCH3	H	H	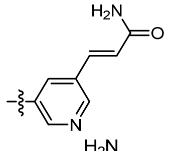	0.145	P
3*	H	-OCH3	H	H	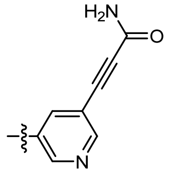	0.153	P
4	H	-OCH3	H	H	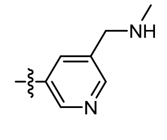	0.065	P

5	H	-OCH3	H	H	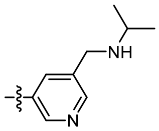	0.375	P
6	H	-OCH3	H	H	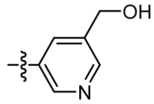	0.208	P
7	H	-OCH3	H	H	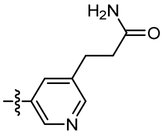	0.162	P
8	H	-F	H	H	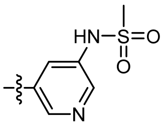	5.64	N
9	H	-F	H	H	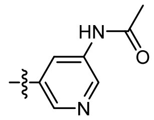	3.68	N
10	H	-OH	H	H	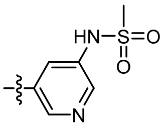	0.28	P
11	H	-OCH3	H	H	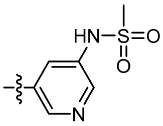	0.26	P
12	H	-OCH3	H	H	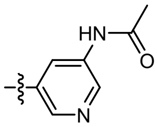	0.04	P
13	H	-OCH3	H	H	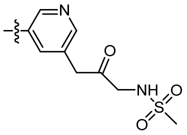	0.491	P
14	H	-OCH3	H	H	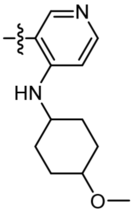	0.207	P
15	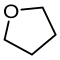		H	H	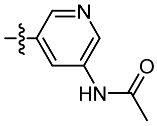	0.644	P
16	H	-OCH3	H	H	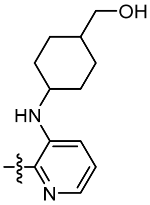	0.33	P
17	H	-OCH3	H	H	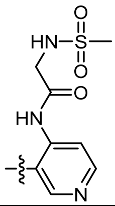	0.0437	P

18	H	-OCH3	H	H	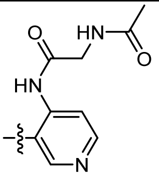	0.281	P
19	H	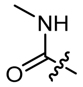	H	H	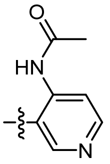	0.593	P
20	H	-OCH3	H	H	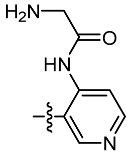	5.42	N
21*	H	-OCH3	H	H	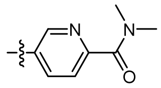	0.175	P
22*	H	-OCH3	H	H	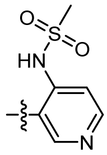	0.922	P
23	H	-OCH3	H	H	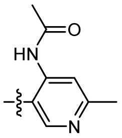	0.15	P
24*	H	-OCH3	H	H	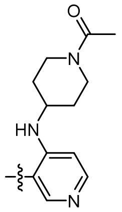	0.0938	P
25	H	-OCH3	H	H	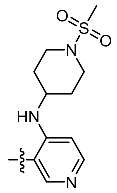	0.0518	P
26	H	-OCH3	H	H	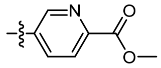	0.35	P
27	H	-OCH3	H	H	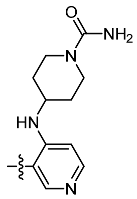	0.644	P
28	H	-OCH3	H	H	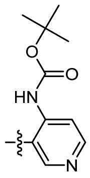	0.171	P
29	H	-CN	H	H	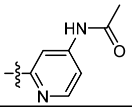	0.487	P

30	H	-OCH$_3$	H	H	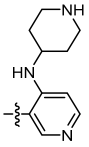	0.637	P
31*	H	-OCH$_3$	H	H	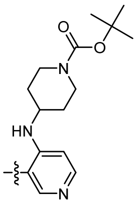	0.789	P
32	H	-OCH$_3$	H	H	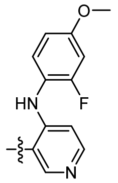	0.351	P
33*	H	-OCH$_3$	H	H	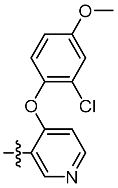	3.86	P
34	H	-OCH$_3$	H	H	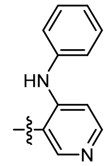	0.808	P
35	H	-OCH$_3$	H	H	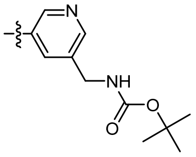	0.413	P
36	H	-OCH$_3$	H	H	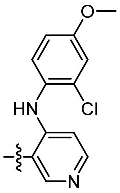	0.534	P
37	H	-OH	H	H	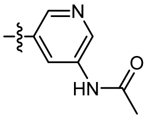	0.658	P
38*	H	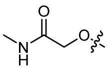	H	H	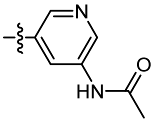	0.0774	P
39	H	-OCH$_3$	H	H	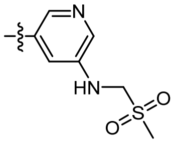	0.488	P
40	H	-OCH$_3$	H	H	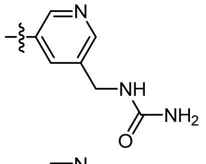	0.273	P
41	H	-OCH$_3$	H	H	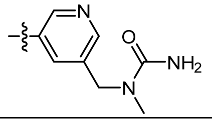	0.236	P

42	H	$-OCH_3$	H	H	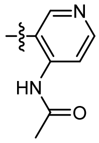	0.247	P
43	H	$-OCH_3$	H	H	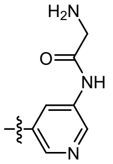	0.337	P
44	H	$-OCH_3$	H	H	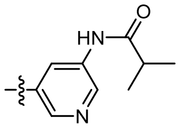	0.904	P
45	H	$-OCH_3$	H	H	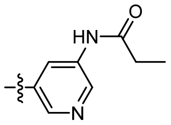	0.306	P
46	H	H	-OH	H	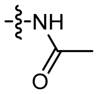	28.1	N
47	H	-OH	H	H	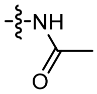	0.8	P
48	-OH	H	H	H	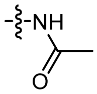	35	N
49	H	H	$-OCH_3$	H	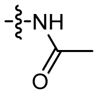	12.3	N
50	H	$-OCH_3$	H	H	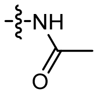	0.4	P
51	$-OCH_3$	H	H	H	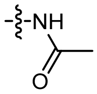	10.5	N
52*	H	H	-F	H	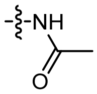	23.6	N
53*	H	-F	H	H	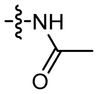	3.9	N
54	H	H	-Br	H	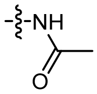	2.3	N
55	H	-Br	H	H	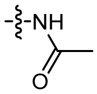	1.1	N
56	H	H	-Cl	H	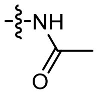	2.9	N
57	H	H	$-CF_3$	H	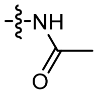	1.1	N
58	H	$-CF_3$	H	H	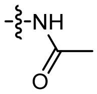	26.5	N
59	H	H	-CN	H	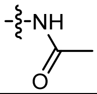	1.3	N

60*	H	H	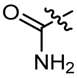	H	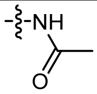	15.7	N
61*	H	H	$-OCF_3$	H	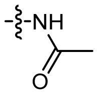	3.2	N
62	H	H	CH_3	H	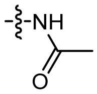	7.3	N
63	H	CH_3	H	H	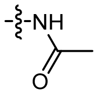	29.3	N
64*	H	H	H	H	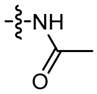	36	N
65	H	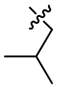	/	/	/	0.043	P
66	H	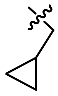	/	/	/	0.109	P
67	H	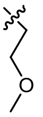	/	/	/	0.272	P
68	H	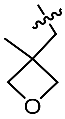	/	/	/	0.315	P
69	H	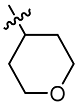	/	/	/	0.0352	P
70	H	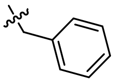	/	/	/	0.106	P
71	H	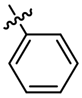	/	/	/	0.013	P
72*	$-NH_2$	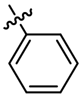	/	/	/	0.0167	P
73	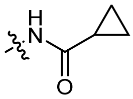	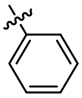	/	/	/	0.301	P
74	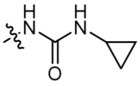	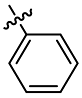	/	/	/	0.015	P
75*	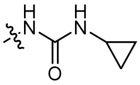	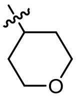	/	/	/	0.0171	P
76*	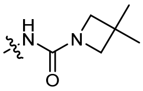	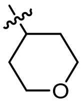	/	/	/	0.0116	P
77	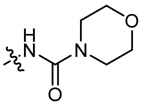	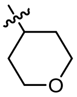	/	/	/	0.0245	P
78	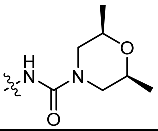	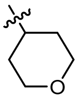	/	/	/	0.0433	P

80*	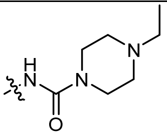	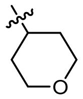	/	/	/	0.0062	P
81	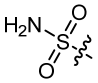	H	/	/	/	0.216	P
82	H	$-NH_2$	/	/	/	0.329	P
83*	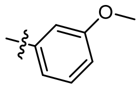	H	H	H	/	0.6	P
84	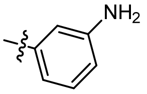	H	H	H	/	0.64	P
85	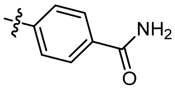	H	H	H	/	0.58	P
86	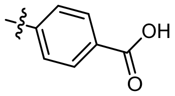	H	H	$-CH_3$	/	0.9	P
87*	-I	H	H	$-CH_3$	/	0.4	P
88*	H	H	-Br	H	/	0.068	P
89	-I	-Br	H	H	/	0.034	P
90	-I	H	-Br	H	/	0.039	P
91	-I	H	H	H	/	0.066	P
92	-OH	H	H	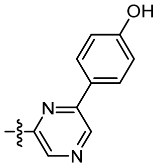	H	0.34	P
93*	H	-OH	H	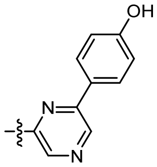	H	0.54	P
95	H	-OH	H	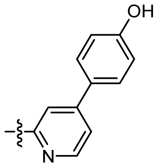	H	0.3	P
96	H	-CN	H	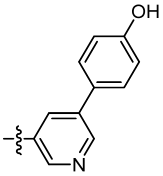	H	1.6	N
97	H	-OH	H	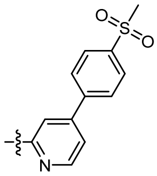	H	0.35	P
98*	-OH	H	H	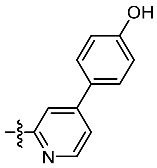	H	0.23	P
99	H	H	-Cl	H	-CN	3.3	N
100*	H	H	-Br	H	-CN	1.1	N

101	H	H	-I	H	-CN	0.41	P
102	H	H	H	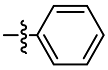	-CN	0.4	P
103*	H	H	-Cl	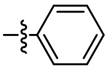	-CN	0.04	P
104*	H	H	-Br	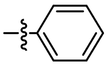	-CN	0.025	P
105	H	H	-I	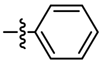	-CN	0.01	P
106*	H	H	-Cl	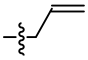	-CN	0.69	P
107*	H	H	-Cl	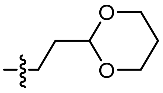	-CN	3.9	N
108	H	H	-I	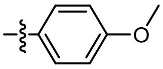	-CN	0.233	P
109	H	H	-I	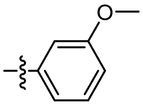	-CN	0.21	P
110*	H	H	-I	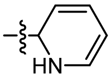	-CN	0.08	P
111	H	H	-I	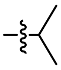	-CN	0.28	P
112	H	H	-I	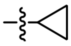	-CN	0.14	P
113	H	H	-I	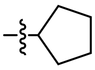	-CN	0.07	P
114	H	H	-Br	H	-CN	1.9	N
115	H	H	-Br	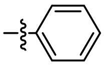	-CN	2.3	N
116	H	H	-I	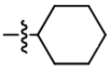	-CN	0.65	P
117*	H	H	-Cl	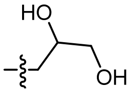	-CN	2	N

[1] "*" is a test set of 29 compounds randomly selected.

Table S2. Structure and activity of DYRK1A inhibitors for the external validation set.

No.	Com.	IC_{50}(μM)	P/N
118	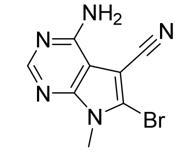	128	N
119	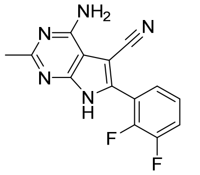	0.068	P
120	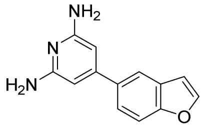	0.014	P
121	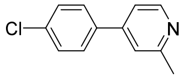	>10	N
122	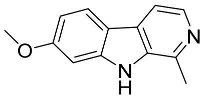	0.08	P
123	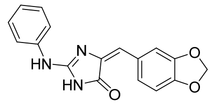	0.032	P
124	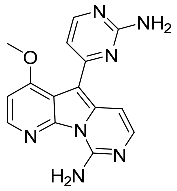	0.08	P
125	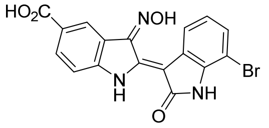	0.21	P
126	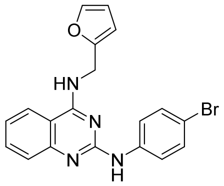	5	N
127	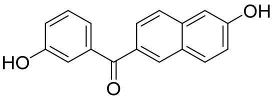	2.9	N
128	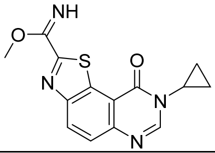	0.091	P
129	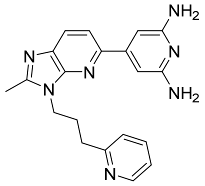	0.006	P

130	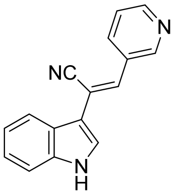	5.5	N
131	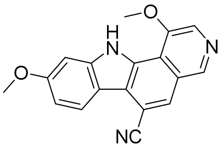	0.018	P
132	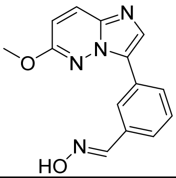	0.033	P

Table S3. Performance of 35 classification models for the training set and test set.

Data set		Model	AUC	CA	MCC	TP	TN	FP	FN	SE	SP
Training set	Ext FP	SVM	0.580	0.773	0.449	58	6	13	1	0.983	0.316
		Neural Network	0.631	0.727	0.163	58	6	13	11	0.841	0.316
		kNN	0.660	0.739	0.113	61	4	15	8	0.884	0.211
		Naïve Bayes	0.663	0.659	0.280	45	13	6	24	0.652	0.684
		Random Forest	0.693	0.750	0.171	61	5	14	8	0.884	0.263
		Logistic Regression	0.676	0.727	0.163	58	6	13	11	0.841	0.316
		Tree	0.626	0.716	0.145	57	6	13	12	0.826	0.316
Training set	EState FP	SVM	0.883	0.852	0.508	67	8	11	2	0.971	0.421
		Neural Network	0.893	0.852	0.556	63	12	7	6	0.913	0.632
		kNN	0.820	0.852	0.516	66	9	10	3	0.957	0.474
		Naïve Bayes	0.846	0.750	0.396	53	13	6	16	0.768	0.684
		Random Forest	0.882	0.852	0.540	64	11	8	5	0.928	0.579
		Logistic Regression	0.882	0.852	0.540	64	11	8	5	0.928	0.579
		Tree	0.843	0.83	0.547	59	14	5	10	0.855	0.737
Training set	MACCS FP	SVM	0.879	0.898	0.678	67	12	7	2	0.971	0.632
		Neural Network	0.872	0.886	0.653	65	13	6	4	0.942	0.684
		kNN	0.865	0.898	0.684	66	13	6	3	0.957	0.684
		Naïve Bayes	0.857	0.716	0.406	48	15	4	21	0.696	0.790
		Random Forest	0.900	0.898	0.678	67	12	7	2	0.971	0.632
		Logistic Regression	0.875	0.875	0.624	64	13	6	5	0.928	0.684
		Tree	0.835	0.875	0.612	65	12	7	4	0.942	0.632
Training set	PubChemFP	SVM	0.933	0.909	0.717	67	13	6	2	0.971	0.684
		Neural Network	0.893	0.909	0.743	64	16	3	5	0.928	0.842
		kNN	0.861	0.861	0.612	65	12	7	4	0.942	0.632
		Naïve Bayes	0.908	0.807	0.508	57	14	5	12	0.826	0.737
		Random Forest	0.908	0.920	0.753	68	13	6	1	0.986	0.684
		Logistic Regression	0.904	0.920	0.755	67	14	5	2	0.971	0.737
		Tree	0.754	0.864	0.583	64	12	7	5	0.928	0.632
Training set	Sub FP	SVM	0.886	0.852	0.515	69	6	13	0	1.000	0.316
		Neural Network	0.908	0.841	0.530	62	12	7	7	0.899	0.632
		kNN	0.862	0.830	0.435	65	8	11	4	0.942	0.421
		Naïve Bayes	0.875	0.773	0.455	54	14	5	15	0.783	0.737
		Random Forest	0.88	0.841	0.513	63	11	8	6	0.913	0.579
		Logistic Regression	0.914	0.864	0.583	64	12	7	5	0.928	0.632
		Tree	0.896	0.875	0.638	63	14	5	6	0.913	0.737
Test set	Ext FP	SVM	0.622	0.724	0.262	19	2	7	1	0.950	0.222
		Neural Network	0.833	0.724	0.286	18	3	6	2	0.900	0.333
		kNN	0.731	0.759	0.380	19	3	6	1	0.950	0.333
		Naïve Bayes	0.772	0.690	0.344	14	6	3	6	0.700	0.667
		Random Forest	0.767	0.759	0.380	19	3	6	1	0.950	0.333
		Logistic Regression	0.800	0.759	0.380	19	3	6	1	0.950	0.333
		Tree	0.703	0.759	0.393	18	4	5	2	0.900	0.444
Test set	EState FP	SVM	0.892	0.724	0.318	17	4	5	3	0.850	0.444
		Neural Network	0.858	0.793	0.517	17	6	3	3	0.850	0.667
		kNN	0.894	0.828	0.596	20	4	5	0	1.000	0.444
		Naïve Bayes	0.786	0.793	0.517	17	6	3	3	0.850	0.667
		Random Forest	0.903	0.759	0.393	18	4	5	2	0.900	0.444
		Logistic Regression	0.831	0.828	0.587	18	6	3	2	0.900	0.667

		Tree	0.731	0.759	0.393	18	4	5	2	0.900	0.444
Test set	MACCS FP	SVM	0.906	0.862	0.680	20	5	4	0	1.000	0.556
		Neural Network	0.911	0.862	0.678	18	7	2	2	0.900	0.778
		kNN	0.856	0.862	0.680	20	5	4	0	1.000	0.556
		Naïve Bayes	0.822	0.759	0.454	16	6	3	4	0.800	0.667
		Random Forest	0.922	0.862	0.680	20	5	4	0	1.000	0.556
		Logistic Regression	0.906	0.897	0.753	19	7	2	1	0.950	0.778
		Tree	0.933	0.862	0.680	20	5	4	0	1.000	0.556
Test set	PubChemFP	SVM	0.911	0.862	0.705	17	8	1	3	0.850	0.889
		Neural Network	0.911	0.862	0.705	17	8	1	3	0.850	0.889
		kNN	0.872	0.897	0.761	20	6	3	0	1.000	0.667
		Naïve Bayes	0.881	0.828	0.647	16	8	1	4	0.800	0.889
		Random Forest	0.917	0.897	0.761	20	6	3	0	1.000	0.667
		Logistic Regression	0.944	0.862	0.705	17	8	1	3	0.850	0.889
		Tree	0.878	0.828	0.647	16	8	1	4	0.800	0.889
Test set	Sub FP	SVM	0.858	0.759	0.393	18	4	5	2	0.900	0.444
		Neural Network	0.881	0.793	0.517	17	6	3	3	0.850	0.667
		kNN	0.853	0.862	0.680	20	5	4	0	1.000	0.556
		Naïve Bayes	0.892	0.759	0.496	15	7	2	5	0.750	0.778
		Random Forest	0.850	0.828	0.577	19	5	4	1	0.950	0.556
		Logistic Regression	0.903	0.793	0.493	18	5	4	2	0.900	0.556
		Tree	0.825	0.862	0.517	17	6	3	3	0.850	0.667

Table S4. PubChem fingerprints of inhibitors (16) and non-inhibitors (10) responsible for DYR1KA modulation/inhibition.

Fingerprints	Substructure	General Sub-structure	Representative Substructure	IG	FP	FN
PubchemFP187	≥2 saturated or aromatic nitrogen-containing ring size 6	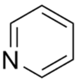	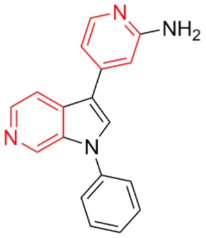	0.088	1.315(23)	0(0)
PubchemFP188	≥2 saturated or aromatic heteroatom-containing ring size 6			0.088	1.315(23)	0(0)
PubchemFP260	≥3 hetero-aromatic rings	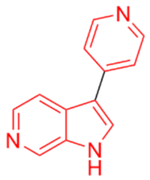	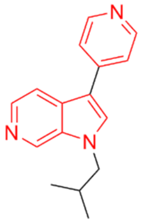	0.067	1.292(18)	0(0)
PubchemFP646	O=C-N-C-[#1]	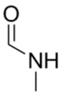	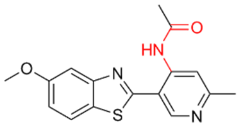	0.063	1.315(17)	0(0)
PubchemFP645	O=C-N-C-C		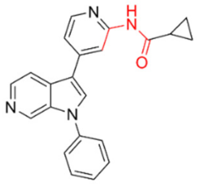	0.053	1.230(29)	0.270(2)

PubchemFP499	N-C:C:N		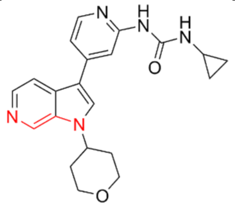	0.064	1.237(32)	0.246(2)
PubchemFP547	N-C:C-N			0.064	1.237(32)	0.246(2)
PubchemFP569	N-C-C-N	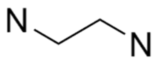		0.060	1.213(36)	0.321(3)
PubchemFP611	N-C-C-N-C			0.060	1.213(36)	0.321(3)
PubchemFP629	S-C:C:C-N	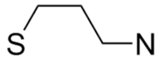	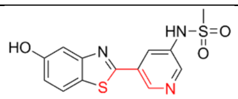	0.062	1.198(41)	0.371(4)
PubchemFP658	C-C-S-C-C	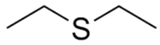	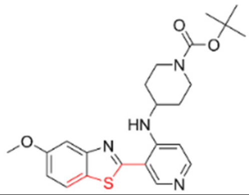	0.062	1.198(41)	0.371(4)
PubchemFP691	O-C-C-C-C-C-N			0.144	1.263(49)	0.164(2)
PubchemFP702	O-C-C-C-C-C-N-C	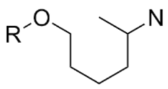	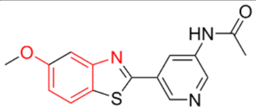	0.144	1.263(49)	0.164(2)
PubchemFP703	O-C-C-C-C-C(N)-C			0.139	1.262(48)	0.167(2)
PubchemFP720	Oc1ccc(S)cc1		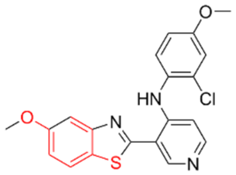	0.103	1.253(41)	0.194(2)
PubchemFP783	OC1CCC(S)CC1	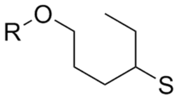		0.103	1.253(41)	0.194(2)
PubchemFP24	≥2F	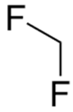	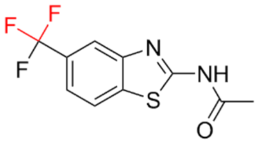	0	0(0)	4.179(3)
PubchemFP363	C(~F)(~F)			0	0(0)	4.179(3)
PubchemFP716	Cc1ccc(N)cc1	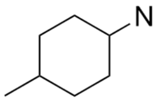	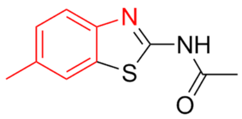	0	0(0)	4.179(4)
PubchemFP505	Cl-C:C-O		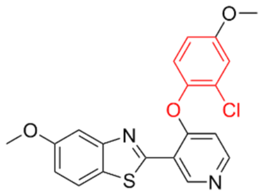	0	0(0)	4.179(1)
PubchemFP583	Cl-C:C-O-C	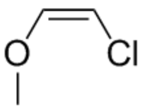		0	0(0)	4.179(1)
PubchemFP827	OC1C(Cl)CCC C1	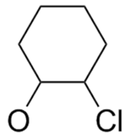		0	0(0)	4.179(1)
PubchemFP347	C(~C)(~H)(~O) (~O)		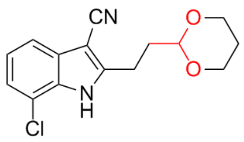	0	0(0)	4.179(1)
PubchemFP367	C(~H)(~O)(~O)			0	0(0)	4.179(1)
PubchemFP727	Sc1ccc(Br)cc1		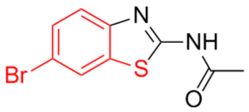	0	0(0)	4.179(1)
PubchemFP811	SC1CC(Br)CCC1			0	0(0)	4.179(1)
